# Early noninvasive prenatal paternity testing by targeted fetal DNA analysis

**DOI:** 10.1038/s41598-023-39367-0

**Published:** 2023-07-26

**Authors:** Géraldine Damour, Karine Baumer, Hélène Legardeur, Diana Hall

**Affiliations:** 1 Unité de Génétique Forensique, Centre Universitaire Romand de Médecine Légale, Centre Hospitalier Universitaire Vaudois et Université de Lausanne, Ch. de Vulliette 4, 1000 Lausanne, Switzerland; 2 Woman-Mother-Child Department, Lausanne University Hospital, Lausanne, Switzerland

**Keywords:** Genetics, Health care

## Abstract

Today the challenge in paternity testing is to provide an accurate noninvasive assay that can be performed early during pregnancy. This requires the use of novel analytical methods capable of detecting the low fraction of circulating fetal DNA in maternal blood. We previously showed that forensic compound markers such as deletion/insertion polymorphisms-short tandem repeats (DIP-STR) can efficiently resolve complex mixed biological evidence including the target analysis of paternally inherited fetal alleles. In this study, we describe for the first time the validation of this type of markers in the first trimester of pregnancies, in addition to defining the statistical framework to evaluate paternity. To do so, we studied 47 DIP-STRs in 87 cases, with blood samples collected throughout gestation starting from the seven weeks of amenorrhea. Fetal DNA detection in the first trimester shows a false negative rate as low as 6%. The combined paternity index (CPI) results indicate that seven markers with fully informative genotypes are sufficient to determine the paternity. This study demonstrates that DIP-STR markers can be used from early pregnancy and that a small set of markers (about 40) is sufficient to address the question of paternity. The novel method offers substantial improvements over similar approaches in terms of reduced number of markers, lower costs and increased accuracy.

## Introduction

In prenatal care, technical advances in the analysis of fetal DNA circulating in maternal blood (cffDNA)^1^ have enabled great progress in early noninvasive prenatal screening and diagnosis of several genetic conditions^2–11^. These advances could also contribute to the handling of difficult situations in forensic science, such as the question of paternity during early pregnancy of sexually abused women. A noninvasive test would offer the possibility of avoiding procedures associated to a certain degree of harm to the mother and the fetus (amniocentesis^12–14^ and chorionic villus sampling^15,16^), in addition to addressing the question of paternity as early as six weeks of amenorrhea.

Because the presence of fetal DNA in the blood stream of the mother is due to the continuous remodeling of the placenta, with trophoblast cells undergoing apoptotic events, its analysis presents several challenges to traditional DNA profiling. CffDNA is mostly short (about 160–200 base-pairs) and it represents 5–15% of the total cell free plasma DNA^17–19^, thus generating an in vivo DNA mixture characterized by a large excess of maternal DNA (unbalanced DNA mixture)^20,21^. As the background maternal DNA interferes with the detection of fetal DNA^22^, analytical methods need to be either highly sensitive for DNA mixture deconvolution or specific to the fetal DNA fraction.

Early studies primarily focused on the use of single-nucleotide polymorphisms (SNPs) because of their short size and the possibility of scaling-up by high throughput technologies. First, SNP microarray were employed for large-scale marker analysis (High-throughput SNP array)^23–25^ which compensates for the SNP’s low discrimination power. The advent of massive parallel sequencing (MPS)^26–34^ offered an extremely efficient method for fetal DNA analysis from maternal plasma: the sequencing of thousands to millions of DNA molecules on a genome-wide scale, makes it possible to reveal the minor fetal genetic component against a background of highly homologous maternal DNA.

Yet, several studies indicated that thousands of SNP markers are recommended to achieve a high accuracy as the loss in uninformative SNPs and false negatives due to the low fetal fraction can be high^26,27,29,32,33^. Improved results came with the use of SNP sets selected based on population variability data (356 SNPs) and the use of Unique Molecular Identifiers (UMI) for more reliable genotyping^31^. Finally, in an effort of reducing the number of possibly linked loci and high costs, an MPS-based assay targeting 60 microhaplotypes was also tested^28,35^.

Yet, these studies show that a well-accepted method including careful data interpretation has not been developed. The evaluation of factors influencing the accuracy of the results, such as degree of marker polymorphism and number for informativeness (maximize number but lowering incidental findings), sequencing depth for a sufficient signal-to-noise ratio, thresholds of fetal fraction as well as sequencing strategy is still ongoing, including validation of the method on a large clinical sample set from early stages of pregnancy.

Alternatives approaches aiming at reducing the recurrent problem of low signal-to-noise ratio propose the targeting the fetal DNA with allele-specific amplifications. These include methylation markers^36^ and more frequently compound markers such as SNP-SNPs^37^ and DIP-STRs^38,39^.

**Figure 1:**
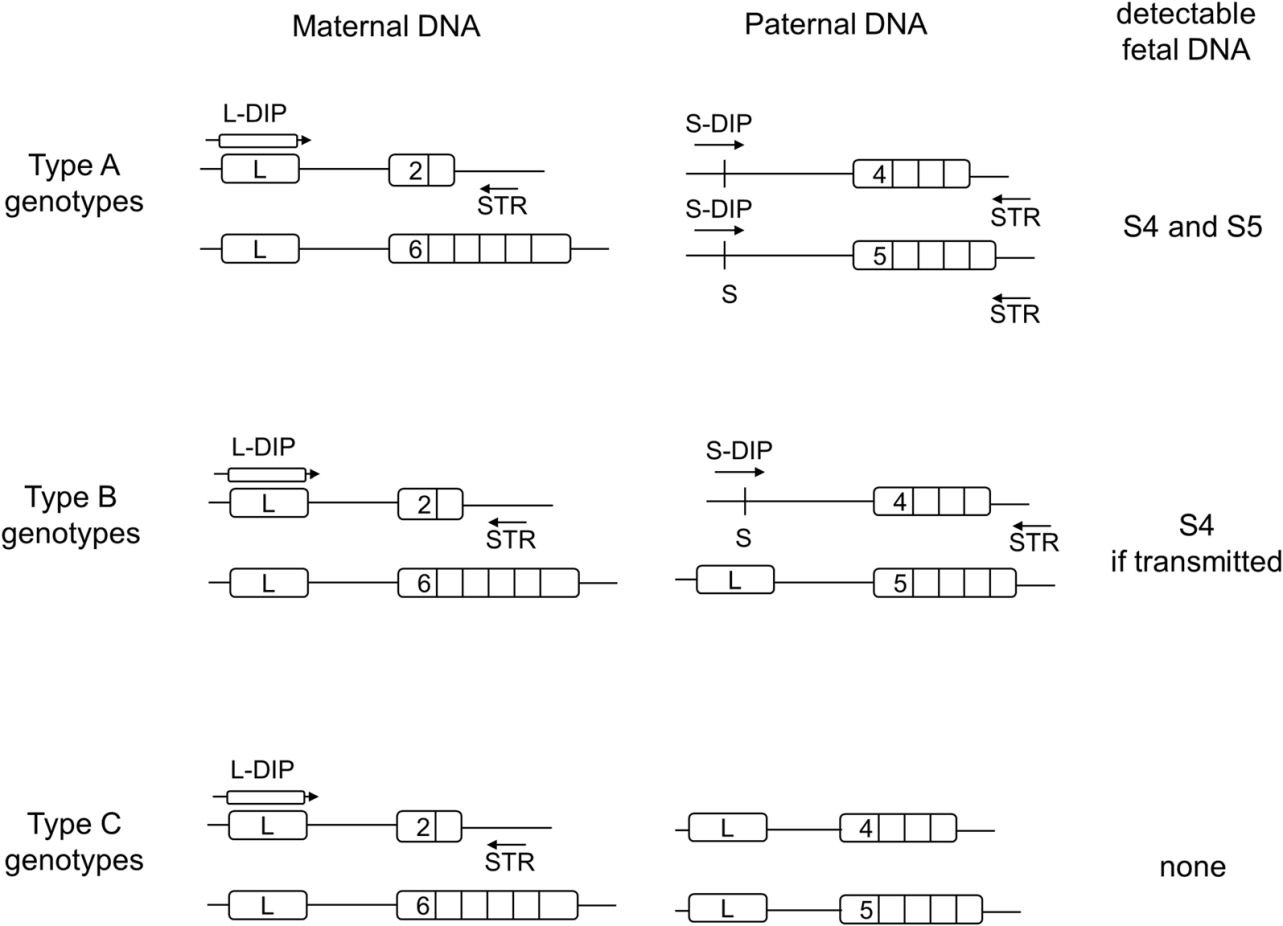
Types of informative DIP-STRs. Type A genotypes, the mother and the father are homozygous for different DIP alleles. With this marker, the paternally transmitted DIP-STR haplotype can be targeted in maternal plasma using allele-specific primers. Type B genotypes, the mother is DIP homozygous and the father is DIP heterozygous. With this marker, the paternally transmitted DIP-STR haplotype can be targeted in maternal plasma if the transmitted haplotype carries a DIP allele not shared with the mother. Type C genotypes, the mother and the father are homozygous for the same DIP allele. With this marker, no paternally transmitted DIP-STR haplotype can be specifically targeted, yet paternity inconsistency are readily identified if the biological father is of either type A or of type B with transmission of the DIP allele not shared with the mother.

DIP-STR markers can resolve extremely unbalanced two-source DNA mixtures of same-or-opposite sex donors, up to a 1:1000 minor:major DNA ratio. Positive results were obtained in targeting DNA sequences unique to the fetal DNA transmitted by the father. These sequences are biallelic deletion/insertion polymorphisms (DIP) of several nucleotides located physically very close to the STR marker, for combined analysis. The multiallelic haplotype composed of both DIP and STR alleles is analyzed by using PCR primers overlapping the deleted/inserted sequence (S-DIP, L-DIP primers) on one side and downstream the STR region on the other side (STR primer) (Fig. 1). A forensic set of 10 markers was validated for casework^40^ and a larger set of 28 DIP-STRs showed an efficient detection of fetal DNA in the plasma of 48 women, whose blood was collected in advanced pregnancy^41^. DIP-STR markers were also used to test zygosity of twin pregnancies^42^. These results show that DIP-STR can be used as a supplementary method especially when cffDNA accounts for less than 10% of total cell free DNA.

In this study, we aimed at improving the number of markers validated for prenatal paternity testing on a large number of cases all collected early in gestation. We studied a set of 47 DIP-STRs in 87 pregnancies, with blood samples collected throughout pregnancy starting from the first trimester. Here, we also provide the general framework for the paternity index calculation with an evaluation of the minimum number of informative markers necessary for determining paternity.

## Results

### Type of informative markers

As described above, the analysis of circulating fetal DNA is based on allele-specific amplifications of the paternally transmitted DIP-STR haplotype when its DIP allele is not shared with the maternal DNA (Fig. 1).

To do so, primers for cffDNA analysis are selected based on the DIP genotype of the mother. Markers informative for the genotypes of the fetus are always DIP homozygous in the mother (SS or LL) and they are analyzed using primers specific to the opposite DIP allele (L- and S-primers, respectively). The fetal allele is then targeted if a DIP-STR haplotype containing a DIP allele different from the mother is transmitted by the father. Considering all possible genotype assortments, three types of informative markers exist (Fig. 1): (i) markers of type A, the father is homozygous for the other DIP allele than the one of the mother. In this case, the paternal haplotype can be targeted in cffDNA analysis; (ii) markers of type B, the father is heterozygous at the DIP locus, in this case the cffDNA can be targeted only if the paternally transmitted haplotype carries the DIP allele different from the mother; finally, (iii) markers of type C, the father is homozygous for the same DIP allele of the mother, in this case no paternal haplotype can be targeted in maternal plasma. Any DIP-STR result inconsistent with the expected genotype of the alleged father, including any positive results from markers of type C, would support the exclusion of the alleged father.

In Table 1 is reported the average number of informative marker observed in the cases presented. When using the set of 47 DIP-STRs the number of parental genotypes of type A are about five and the number of either type B or C is 11–12, with a total of about 28 informative markers available for each family to determine the paternity.

**Table 1:** The average number of informative DIP-STRs per family using a set of 47 markers in 87 cases.

Marker	Mean ± SD
Type A	5 ± 2
Type B	11 ± 3
Type C	12 ± 3
Total	28 ± 3

### Performance of DIP-STR specific amplification of cffDNA

Singleplex genotyping of markers of type A was done on selected DIP-STRs with the aim of testing their performance in the first trimester across cases. The consumption of cffDNA was optimized to enable the testing of several markers per case and each marker across several cases. Moreover, to further control for PCR specificity, the priority was given to the genotyping of cases with fathers heterozygous for the STR. Samples from the second and third trimester were used for confirmation.

**Figure 2:**
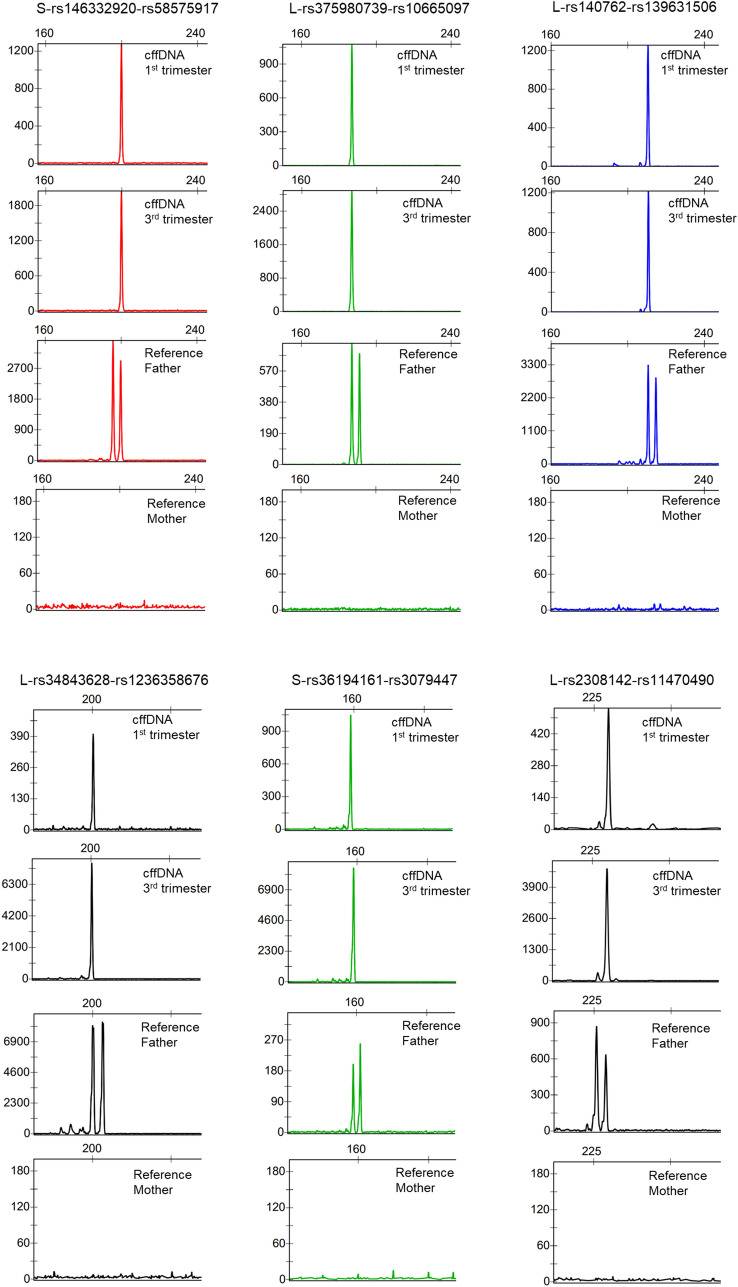
Examples of fetal DNA detection in maternal plasma with DIP-STR markers. Electropherogram results of the positive detection of the paternally transmitted alleles in maternal blood collected during the first and third trimester. The detected allele can be compared to the parental genotypes indicated below. The STR heterozygosity of the father allowed us to confirm the target amplification of the paternal haplotype of the fetus. Primers are selected to be specific to the paternally transmitted alleles. Therefore, as expected, no PCR product is detected when using the reference DNA of the mother.

**Figure 3:**
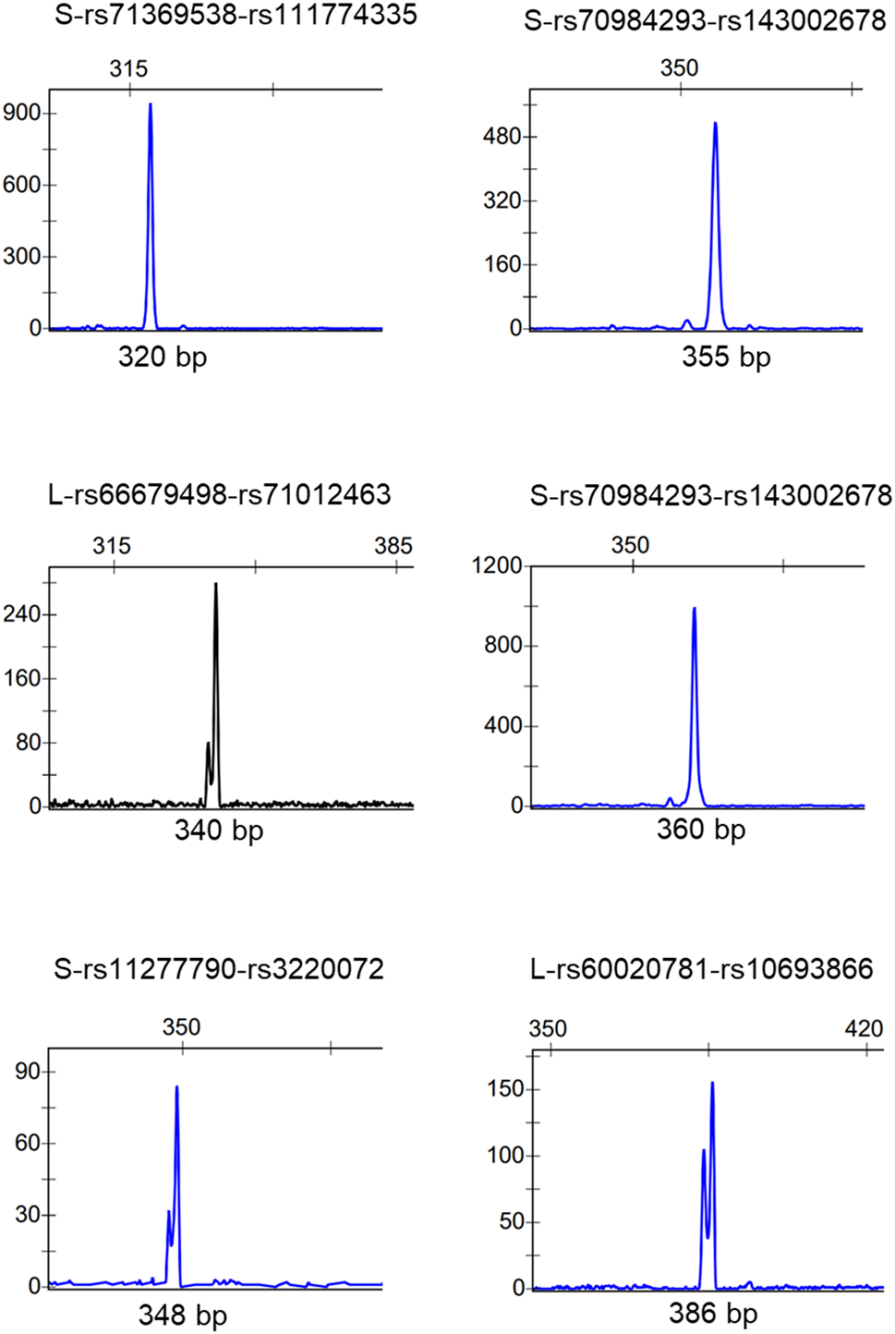
Examples of fetal DNA detection in maternal plasma with long DIP-STR markers. Electropherogram results of the positive detection of the paternally transmitted alleles in maternal blood using long DIP-STR markers. The base-pairs (bp) of the detected allele are indicated on the x axis.

The electropherograms of few representative samples are illustrated in Fig. 2 in addition to examples of positive results from the longest DIP-STRs of our collection (Fig. 3).

We didn’t observe a difference between standard EDTA and Streck Cell-Free DNA BCT^®^ collection tubes in the success rate of cffDNA amplification.

All cases of amplification failure of fetal DNA clustered in the first trimester, with 10 false negative cases out of 164 fetal allele interrogated (Table 2). This false negative rate is of about 6% in the first trimester and close to zero in samples from second and third trimester. Eight markers longer than 300 bp were not included in this estimate, as well as the marker rs71070706-rs147416097 that is not sensitive enough and should be eliminated for clinical applications. The markers associated to these weak and negative results are all characterized by low PCR efficiency as previously reported, Refs.^38,39,43^ these are L-rs55886629-rs56078928, L-rs59055342-rs71557834, L-rs61437086-rs140235473, S-rs71725104-rs10656000, S-rs138331044-rs113027169, S- and L-rs145299629-rs200177067. No false positive amplification of maternal alleles were observed except for few markers L-rs56821990-rs200925554, S- and L-rs3216342-rs10639027, S-rs61437086-rs140235473, S-rs140762-rs139631506, S-rs552898832-rs58403232, which produce extra peaks 100/200 bp far from the expected signal of constant size, each time an excess of DNA is used.

**Table 2:** DIP-STR positive results in the 1st trimester.

DIP-STR	S-DIP-STR	L-DIP-STR
rs57158370-rs59980295	2/2	1/1
rs140348786-rs78039244	2/2	1/1
rs70984293-rs143002678*	nt	nt
rs34079143-rs58766997	1/1	1/1
rs145299629-rs200177067	1/2	1/3
rs56821990-rs200925554	1/1	3/3
rs57312079-rs33940604	2/2	1/1
rs34447739-rs60404498	1/1	2/2
rs10550804-rs10626303	1/1	3/3
rs148778359-rs59509704*	nt	nt
rs146524520-rs10595212	1/1	2/2
rs71369538-rs111774335*	1/1	0/1
rs6144148-rs71032506	1/1	4/4
rs59855564-rs10552735	2/2	3/3
rs111312404-rs146792075	1/1	1/1
rs139619099-rs71122692	1/1	1/1
rs11282651-rs57316542	1/1	1/1
rs71113068-rs60126987	1/1	1/1
rs61345556-rs57072260	2/2	1/1
rs3216342-rs10639027	1/1	1/1
rs56348349-rs60422854	2/2	4/4
rs55886629-rs56078928	2/2	1/2
rs113508481-rs11466859	1/1	2/2
rs35032587-rs35006796	4/4	2/2
rs10533007-rs138121885	2/2	1/1
rs375980739-rs10665097	1/1	8/8
rs59055342-rs71557834	2/2	0/2
rs61437086-rs140235473	4/6	2/2
rs146332920-rs58575917	2/2	0/0
rs71070706-rs147416097	nt	nt
rs34843628-rs1236358676	4/4	3/3
rs145423446-rs34420283	5/5	4/4
rs2308142-rs11470490	2/2	3/3
rs1611095-rs56730261*	1/3	2/3
rs11277790-rs3220072*	1/1	0/1
rs60194384-rs59823002*	1/2	0/3
rs60020781-rs10693866*	0/1	1/1
rs66679498-rs71012463*	0/2	1/2
rs71725104-rs10656000	3/4	5/5
rs11279993-rs780572076	2/2	1/1
rs139592446-rs4001398	2/2	1/1
rs36194161-rs3079447	1/1	1/1
rs138331044-rs113027169	2/3	1/1
rs140762-rs139631506	5/5	5/5
rs3054057-rs3054059	3/3	4/4
rs11278940-rs59122733	1/1	1/1
rs552898832-rs58403232	1/1	4/4

Results are expressed as positive results over number of tested (n/n).

*nt* not tested.

*Markers longer than 300 bp not included on the false negative estimate.

### Paternity determination

**Figure 4:**
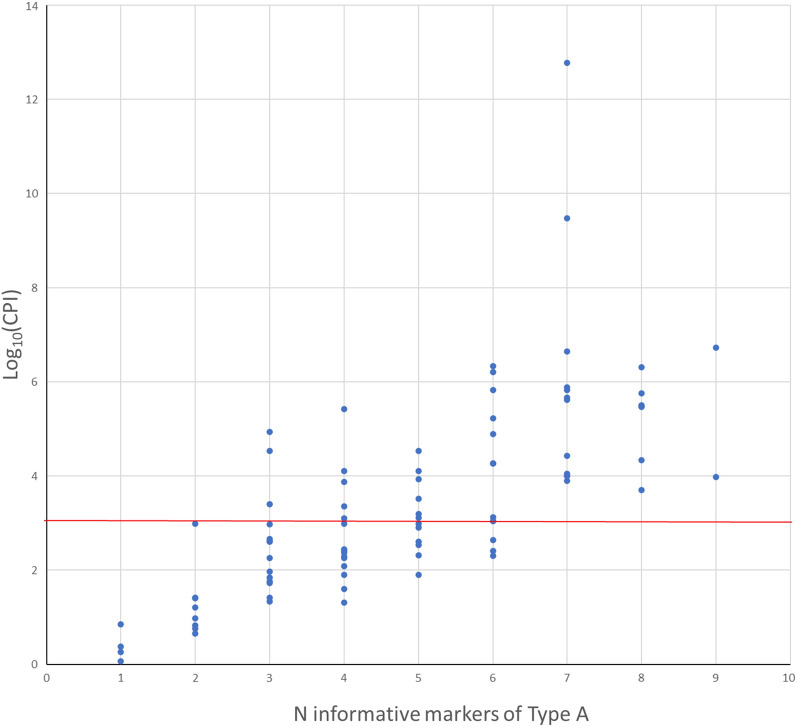
Log_10_(CPI) values of the alleged fathers for the 87 cases. Values were calculated considering only markers presenting genotypes of type A. Cases were grouped according to the number of informative markers of type A analyzed. The red line indicates the value of Log_10_(CPI) of 3, corresponding to a probability of paternity of 99.9%, which is the value accepted in most country for the verbal conclusion about practically proven paternity.

After the initial screening of the 47 DIP genotypes of the parents, the complete transmitted DIP-STR haplotype was determined only for markers of type A. Therefore, the PI calculated for each case is based on genotypes of type A. As expected, CPI values increase with the number of informative markers. At least seven markers are necessary to reach a CPI higher than 1,000, which is the value accepted in most country for the verbal conclusion about practically proven paternity. As expected, one or two markers of type A are never sufficient to demonstrate paternity (Fig. 4).

Markers of type C, as markers of type A, are extremely useful for the question of paternity, especially for a quick and clear exclusion of paternity. Markers of type C for the alleged father can be of type A or of type B for the biological father, the associated probabilities of such genotype assortment are (s^4^l^2^) + (l^4^s^2^) + (s^4^2sl) + (l^4^2sl). It should be considered that when the biological father is heterozygous, his transmitted allele can be informative or not. It follows that, considering half of the cases where the biological father is DIP heterozygous, the genotypes potentially showing allelic inconsistencies have the following probabilities: (s^4^l^2^) + (l^4^s^2^) + (s^4^sl) + (l^4^sl). This probability is roughly 6.25%. In these cases, positive fetal DNA results are obtained which are not expected based on the alleged father’s genotype. In addition to these cases, markers of type C for the biological father can be of type A or B for the alleged father. They similarly contribute to paternity exclusion: (s^4^l^2^) + (l^4^s^2^) + (s^4^sl) + (l^4^sl). Under these genotypes, the expected fetal DNA results is not observed. This means that, considering balanced S and L allele frequencies, 12.5% of the markers show a genetic inconsistency in cases of non-paternity. It should be noted that, when considering the whole DIP-STR haplotype information, many more markers (about 28 when using a set of 47 markers) provide data for determining paternity (Table 1).

## Discussion

This study demonstrates that DIP-STR markers can be used to detect paternal alleles in cffDNA from seven weeks into pregnancy and that a small set of markers (about 40) is sufficient to address the question of paternity.

Our results are based on a large number of cases, namely 87, all collected during the first trimester with additional samples per case collected in the second and/or third trimester for results confirmation. This enabled us to have for each marker at least one informative case and possibly several. It should be noted, that cffDNA accounts for approximately 5–20% of the total cffDNA, with an upward trend evident through pregnancy. Previous studies indicate the fetal fraction in the 1^st^ trimester as low as 3–4% in 30% of the cases^26,28^. It is therefore essential to test the feasibility and accuracy of the method in real cases and early during pregnancy. Recent reports of similar studies either didn’t include samples from the first trimester^27,37,44^ or included about 10 cases^28–33^. This doesn’t allow to have all markers informative in at least one case and therefore evaluate marker specific false positive/negative rate which may vary.

In our study, we tested all DIP-STR markers available, including long ones (> 300 bp) and less specific/sensitive to produce empirical data for marker exclusion. The failure rate of each marker in the first trimester was estimated on a selection of cases showing informative genotypes of type A, with parents opposite homozygous for the DIP alleles. Here, the false negative rate reached 6%. Interestingly, markers longer than 320 bp to 386 bp worked in 8/22 PCR assays, yet we didn’t include them in the estimate of the false negative rate. The partial success with much longer markers is probably due to the allele-specificity of the method which enables the use of the much reduced fetal DNA fraction that is longer than the average apoptotic DNA fragments. If necessary, long DIP-STR can be used for cffDNA detection if results are interpreted using the related increased drop-out probability. Few observed false positive signals were limited to extra peaks far from the expected allele size that could be easily excluded with the GeneMapper^®^ software’s bin set system for scoring the data. We didn’t observe a difference between standard EDTA and Streck Cell-Free DNA BCT^®^ collection tubes in the quality and/or quantity of cffDNA. This may be due to the fact that the Streck Cell-Free DNA BCT^®^ collection tubes were still stored at 4 °C immediately after collection and sample processing was never delayed longer than four hours.

Finally, the comparison of false negative rates of DIP-STRs to other markers was limited by the reduced number of studies including a sufficient number of first trimester cases. When these cases are included, often results are pooled for the whole sample collection including all time points. Several studies by Ou’s group employing the MPS technology reported false negative rates of 20–50%^29,32,33^. The lowest reported values are around 15% when using microhaplotypes and fetal fractions estimated at less than 5%^28^.

With the DIP-STR markers, the molecular approach employed for DNA mixture deconvolution assures a fetal specificity that is superior to any currently proposed solution. Interestingly, the minor DNA specificity achieved by combining two types of polymorphisms also contributes to the enhanced informativeness of the markers. In previous studies, the use of SNPs associated with MPS showed that a larger number of SNPs compared with STRs, needed to be interrogated to compensate for the lower discrimination power. About 50 SNPs, having allele frequencies between 0.2 and 0.8, are equivalent to the use of 12 STRs in postnatal tests^45^. In terms of variability, DIP biallelic polymorphisms are expected to perform as SNPs, yet the haplotypes from combining STR variants are associated with much smaller allele frequencies and higher discrimination power.

Moreover, MPS SNP genotyping without a method for targeting the minor DNA, generates noisy data resulting from low fetal allele concentrations, against which the analysis of more SNPs is required to allow for filtering low-quality data. Deep sequencing results require an effective analysis algorithm which can indeed be developed, yet it is still not clear whether a routine high-throughput service laboratory would have a standard analysis pipeline that doesn’t need to be adjusted depending on the efficiency of each experiment, read coverage and accuracy of the estimated fetal fraction.

It is worth emphasizing that our method currently based on PCR-CE, is compatible with a procedure that includes screening of informative markers by DIP multiplex genotyping of the reference DNA (mother and alleged father) followed by singleplex genotyping of selected informative markers. To make use of all the genetic data and avoid the risk of insufficient markers for paternity determination it is necessary to develop two large multiplex for S- and L-allele specific genotyping. Depending on the number of markers selected, the MPS technology may be solution of choice. In this case, the signal to background noise ratio would still be high because of the target enrichment of fetal DNA. Moreover, the results would include all informative and uninformative markers to produce high-confidence fetal genotype calls. The associated Bayesian framework would provide an unsupervised approach convenient for also testing several alleged fathers with one set of data.

However, it should be noted that as few as seven informative markers with parents DIP homozygous for different alleles (markers of type A), were sufficient in our cases to determine paternity. Moreover, at least 12.5% of the genotyped markers are expected to show clear cases of paternity exclusion (unexpected positive or negative fetal DNA amplifications) for an unrelated alleged father. This means that many cases of prenatal paternity testing can be handled with a quick and cost effective solution that uses the technology available in all forensic genetics laboratories. Certainly, markers of type B and the whole DIP-STR haplotype information should still be considered to increase the weight of the evidence.

In conclusion, the current study provides several elements supporting the further use of DIP-STR markers for non-invasive prenatal paternity testing. These are: comprehensive marker set, first trimester validation of all markers, values of false positive and negative rates and a statistical framework for interpretation. This work provides the basis for the forensic development of a standardized prenatal paternity test. Further, deep sequencing based multiplexing is recommended for the improvement of the testing efficiency.

## Methods

### Sample collection

Inclusion criteria for enrolled couples were singleton pregnancies with known paternity. Maternal blood samples (10 ml) were drawn longitudinally from 87 women at 7–13 weeks of amenorrhea (first trimester) (Table 3). For each case, additional blood samples were collected during either the second trimester (14–26 weeks) and/or the third trimester (27–40 weeks). Venous blood samples were drawn into EDTA blood collection tubes and Streck Cell-Free DNA BCT^®^ tubes (Streck, USA). Plasma was separated from the blood cells via double centrifugation (1,600 g for 10 min, tube transfer, and centrifugation at 18,000 g for 10 min) within 2–4 h of blood being drawn. Aliquots of 1 ml were stored at − 20 °C until further processing^46^.

**Table 3:** Gestational age in weeks of amenorrhea for 87 families.

Cases N	Gestational age (wks)
2	7
20	8
21	9
23	10
12	11
4	12
5	13

DNA samples from both parents of the developing baby were collected by buccal swab. The current study was approved by the Centre Hospitalier Universitaire Vaudois and Université de Lausanne institutional review board, research protocol number (2019-01601 CER-VD). Written informed consent was obtained from all participants. All methods were performed in accordance with the relevant guidelines and regulations.

### DNA extraction

Cell free circulating DNA was extracted in duplicate from 2 ml of plasma by using the QIAamp Circulating Nucleic Acid Kit (Qiagen AG, Basel, Switzerland) according to the manufacturer’s protocol. Absorbed DNA was eluted with 60 µl of provided elution buffer. The synthetic DNA RT-SPCY-T02 (Eurogentec, Angers, France) was added to the plasma to function as positive control for circulating DNA extraction. According to the manufacturer’s protocol, 2 ul of a tenfold diluted RT-SPCY-T02 was added to 2 ml of plasma. Reference buccal samples were extracted using the QIAamp DNA Mini Kit (Qiagen AG, Basel, Switzerland) according to the manufacturer’s protocol and eluted in 100 µl final volume. Both genomic and circulating DNA samples were stored at − 20 °C. DNA was extracted using the QIAamp DNA Mini kit (Qiagen AG Switzerland) according to the manufacturer’s guidelines and quantified using the kit QuantiFiler Trio on a QuantStudio™ 5 System (Life Technologies Europe, Zug, Switzerland). The commercial DNAs CEPH 1347-02, Control DNA 007 (Life Technologies Europe, Zug, Switzerland), 2800 M Control DNA (Promega, Dübendorf, Switzerland) were genotyped as a reference controls for allele designation.

### DIP-STR genetic markers

The DIP-STR markers genotyped for this study include 24 markers previously published^38,39,47^ and 23 newly developed^43^ (Table 4). PCR reactions for the markers were performed as previously published DIP-STR genotyping protocols^38,39,43,47^ using 10 ul of cffDNA. S- and L-DIP-STR specific amplifications were done in singleplex according to published protocols^38,39,43,47^. For cffDNA amplifications PCR conditions are modified to increase sensitivity with 36 cycles and twice the amount of PCR primers. To identify informative markers for plasma DNA analysis, reference DNA samples from the mothers and the fathers were first genotyped for 47 DIP markers using seven multiplex reactions as described in Supplementary Table S1 and in Moriot et al. 2019^47^. PCR fragments were separated by capillary electrophoresis after adding 1 μl PCR amplicon to 8.5 μl deionized formamide HI-DI (Life Technologies Europe, Zug, Switzerland) and to 0.5 μl 600 LIZ size standard (Life Technologies Europe, Zug, Switzerland). Capillary electrophoresis was performed using an ABI PRISM 3500*xl* Genetic Analyzer (Life Technologies Europe, Zug, Switzerland) according to the manufacturer's instruction and analyzed using the GeneMapper^®^ ID v3.2.1 software (Life Technologies Europe, Zug, Switzerland), with a minimum peak height threshold of 50 Relative Fluorescence Unit (RFU). The commercial DNA CEPH 1347–02 (Life Technologies Europe, Zug, Switzerland) was used as positive control of amplification and internal standard for allele designations. For standard PCR reactions (28–30 cycles) 0.5 ng of commercial reference DNA was used, for all PCR reactions with increased number of cycles (34–36) 0.0125 ng of commercial reference DNA was used.

**Table 4:** DIP-STR marker list.

DIP-STR	Chr	DIP-STR size (bp)	Haplotype N	Obs. Het	Refs
rs57158370-rs59980295	1p31.1	169–204	13	0.84	^43^
rs140348786-rs78039244	1p36.12	182–220	13	0.80	^43^
rs70984293-rs143002678	1p36.21	347–367	8	0.70	^43^
rs34079143-rs58766997	2p23.1	149–163	5	0.53	^43^
rs145299629-rs200177067	2q23.3	231–252	12	0.71	^43^
rs56821990-rs200925554	3p24.1	209–234	12	0.80	^43^
rs57312079-rs33940604	3q13.33	216–232	8	0.66	^43^
rs34447739-rs60404498	4q35.1	273–318	17	0.90	^43^
rs10550804-rs10626303	5p15.31	201–221	12	0.78	^43^
rs148778359-rs59509704	5q21.2	320–342	10	0.75	^43^
rs146524520-rs10595212	6q16.1	205–216	6	0.51	^43^
rs71369538-rs111774335	9q22.33	320–344	5	0.65	^43^
rs6144148-rs71032506	10q26.2	216–240	11	0.76	^43^
rs59855564-rs10552735	11q22.1	189–196	6	0.78	^43^
rs111312404-rs146792075	11q23.3	176–186	7	0.57	^43^
rs139619099-rs71122692	13q31.1	246–263	8	0.70	^43^
rs11282651-rs57316542	14q24.3	177–188	7	0.70	^43^
rs71113068-rs60126987	15q21.3	228–260	13	0.85	^43^
rs61345556-rs57072260	15q26.1	236–266	10	0.79	^43^
rs3216342-rs10639027	16p12.1	131–208	6	0.55	^43^
rs56348349-rs60422854	17q24.1	245–263	10	0.73	^43^
rs55886629-rs56078928	20p13	184–211	10	0.77	^43^
rs113508481-rs11466859	20q13.12	267–296	15	0.75	^43^
rs35032587-rs35006796	15q26.1	239–271	11	0.88	^39^
rs10533007-rs138121885	2q34	210–234	11	0.64	^39^
rs375980739-rs10665097	7p14.1	182–194	6	0.67	^39^
rs59055342-rs71557834	1q25.3	146–184	15	0.83	^39^
rs61437086-rs140235473	2p25.3	240–260	6	0.65	^39^
rs146332920-rs58575917	9q31.3	175–197	8	0.73	^39^
rs71070706-rs147416097	1p12	222–254	15	0.89	^39^
rs34843628-rs1236358676	4q21.3	182–230	14	0.70	^39^
rs145423446-rs34420283	16p13.2	234–256	11	0.84	^39^
rs2308142-rs11470490	20p13	213–231	10	0.78	^39^
rs1611095-rs56730261	5q23.2	299–345	15	0.49	^38^
rs11277790-rs3220072	10q25.1	340–371	15	0.73	^38^
rs60194384-rs59823002	15q26.2	283–325	12	0.88	^38^
rs60020781-rs10693866	5q11.2	379–395	13	0.62	^38^
rs66679498-rs71012463	2q32.3	331–359	11	0.69	^38^
rs71725104-rs10656000	13q31.3	211–235	7	0.59	^48^
rs11279993-rs780572076	5p13.1	208–236	9	0.65	^48^
rs139592446-rs4001398	2q24.2	154–174	6	0.51	^48^
rs36194161-rs3079447	2q32.1	138–178	13	0.82	^48^
rs138331044-rs113027169	1p12	266–302	18	0.88	^48^
rs140762-rs139631506	6q16.1	179–227	10	0.77	^48^
rs3054057-rs3054059	15q25.3	299–311	7	0.70	^48^
rs11278940-rs59122733	11p13	197–222	3	0.52	^48^
rs552898832-rs58403232	6q14.1	201–226	10	0.32	^48^

*Chr.* Chromosome, *bp* base-pairs, *Obs. Het.* Observed heterozygosity.

### Probability of paternity

The paternity index (PI) was calculated as the ratio of likelihood values of two hypotheses (H0: the test man is the biological father of the child; H1: the test man is unrelated) based on 158 European allele frequency^48^. H0 is equal to 1 if the alleged father is homozygous for the observed DIP-STR haplotype shared with the child, and it is 0.5 when the alleged father is heterozygous for the observed DIP-STR. H1 corresponds to the frequency of all the homozygous and heterozygous individuals of the observed DIP-STR in the populations. H0/H1 = 1/(the frequency of the observed DIP-STR) if the alleged father is homozygous for the DIP-STR and 0.5/(the frequency of the observed DIP-STR) if the alleged father is heterozygous for the DIP-STR). The CPI is the product of the PI of unlinked loci. All marker combinations used for each CPI calculation included unlinked markers either located on different chromosomes or chromosomal arms. Those located on the same chromosomal region were more distant than 6 Mb, on average at about 40 Mb distance, with the exception of two cases at 1.5 Mb and three cases at 0.5 Mb which tested negative for allelic associations in Europe^43^. According to the Swiss national technical specification for parentage testing, inclusion of parenthood is noted when the CPI is greater than 369 which corresponds to a log_10_(CPI) of 2.57.

### Ethics approval

The current study was approved by the Centre Hospitalier Universitaire Vaudois and Université de Lausanne institutional review board, research protocol number (2019–01,601 CER-VD).

### Consent to participate

Each blood sample used was freely donated under conditions of informed consent to participate.

## Supplementary Information

Supplementary Table S1.

## Data Availability

Markers information will be available at https://www.curml.ch/node/65.
