# Supplementary material for: Early noninvasive prenatal paternity testing by targeted fetal DNA analysis
Source: Sci Rep. 2023 Jul 26;13:12139. doi: 10.1038/s41598-023-39367-0 (PMC10372148; doi:10.1038/s41598-023-39367-0)
Supplement: Supplementary file 1 — Supplementary Table S1. [file 41598_2023_39367_MOESM1_ESM.pdf]

Supplementary Table S1

DIP multiplex reactions.

Multiplex group	Marker	DIP primers conc. (nM)
1	rs140348786-rs78039244	100
	rs71369538-rs111774335	400
	rs3216342-rs10639027	400
	rs145299629-rs200177067	400
	rs34447739-rs60404498	200
	rs61345556-rs57072260	400
	rs70984293-rs143002678	300
	rs146524520-rs10595212	400
	rs55886629-rs56078928	400
2	rs11282651-rs57316542	400
	rs34079143-rs58766997	150
	rs111312404-rs146792075	1200
	rs57312079-rs33940604	1600
	rs59855564-rs10552735	200
	rs71113068-rs60126987	
3	rs139619099-rs71122692	200
	rs6144148-rs71032506	400
	rs10550804-rs10626303	200
	rs57158370-rs59980295	800
	rs148778359-rs59509704	200
	rs56348349-rs60422854	200
	rs113508481-rs11466859	400
	rs56821990-rs200925554	1600

Annealing temperature 55°C, 30 cycles.
